# Patients’ experience of important factors in the healthcare environment in oncology care

**DOI:** 10.3402/qhw.v8i0.20870

**Published:** 2013-08-06

**Authors:** Maria Browall, Ingalill Koinberg, Hanna Falk, Helle Wijk

**Affiliations:** 1 Division of Nursing, Department of Neurobiology, Care Sciences and Society, Karolinska Institute, Stockholm, Sweden; 2 School of Life Sciences, University of Skövde, Skövde, Sweden; 3 Institute of Health and Care Sciences, Sahlgrenska Academy, University of Gothenburg, Gothenburg, Sweden; 4 Institute of Neuroscience and Physiology, Sahlgrenska Academy, University of Gothenburg, Gothenburg, Sweden; 5 Institution of Caring Science, Sahlgrenska Academy, Sahlgrenska University Hospital, Gothenburg, Sweden

**Keywords:** Patients' experience, patient perspective, oncological care, physical and psychosocial factors, person-centred care

## Abstract

**Background and objective:**

The aim of this study was to describe what factors of the healthcare environment are perceived as being important to patients in oncology care.

**Design:**

A qualitative design was adopted using focus group interviews.

**Setting and participants:**

The sample was 11 patients with different cancer diagnoses in an oncology ward at a university hospital in west Sweden.

**Results:**

Analysis of the patients’ perceptions of the environment indicated a complex entity comprising several aspects. These came together in a structure consisting of three main categories: safety, partnership with the staff, and physical space. The care environment is perceived as a complex entity, made up of several physical and psychosocial aspects, where the physical factors are subordinated by the psychosocial factors. It is clearly demonstrated that the patients’ primary desire was a psychosocial environment where they were seen as a unique person; the patients wanted opportunities for good encounters with staff, fellow patients, and family members, supported by a good physical environment; and the patients valued highly a place to withdraw and rest.

**Conclusions:**

This study presents those attributes that are valued by cancer patients as crucial and important for the support of their well-being and functioning. The results show that physical aspects were subordinate to psychosocial factors, which emerged strongly as being the most important in a caring environment.

This study focuses on experiences in the healthcare environment among patients affected by cancer. For patients with incurable and possibly life-threatening illnesses, engaging with the complex and often fragmented healthcare system can cause psychological distress (Bakker et al., 2012). Therefore, a crucial factor when creating a person-centred healthcare environment is to support the formation of therapeutic relationships between professionals, patients, and their significant others, based on mutual trust, dignity, understanding, and sharing of human experiences (McCormack & McCance, 2006). These links will then promote an environment that has seamless transitions between levels of care, a high degree of patient and family participation, and an organization that promotes staff satisfaction (Dijkstra, Pieterse, & Pruyn, 2006; Lorenz, 2007; Olsson, Karlsson, & Ekman, 2006; Rashid, 2006; Ulrich & Wilson, 2006).

The importance of measuring patients’ experiences, expectations, and satisfaction with their healthcare environment has long been recognized (Bowling et al., 2012); as early as 1869, Nightingale stressed the impact of physical design on quality of care (Nightingale, 1869/2010). In addition, numerous conceptual models offer a wide array of definitions and insights into the many spatial aspects of nursing and the environmental patterns that promote health within the individual (Andrews & Moon, 2005a, 2005b; Kolanowski, Litaker, & Catalano, 2002). Geographical studies in nursing refocus on the metaconcept of environment in order to gain understanding about how nursing relates dynamically to space and place (Andrews & Shaw, 2008), and how human experiences, behaviour, and activity in relation to health and well-being might influence and represent space and place (Andrews & Phillips, 2005). When considering this as a discipline and research area, it is important not to limit nursing care to solely the nurse–patient relationship, but to also include the therapeutic potential of using the physical and psychosocial environments and their interrelated impacts on personal, societal, and cultural processes (Edvardsson, Sandman, & Rasmussen, 2008; Low & Altman, 1992).

There is great variety in what people expect from healthcare services; for example, being seen in a timely manner; being given adequate advice and information about their condition, symptom management, and side effects; and being involved in their treatment decisions are all common expectations described in earlier research. To meet these expectations, it is important to review these aspects of care from the patient's perspective and to start with knowing the person (Bowling et al., 2012; Edvardsson, Sandman, & Rasmussen, 2006).

A person-centred approached is regarded as synonymous with the best quality of care based on holistic human values (Edvardsson et al., 2008; McCormack, 2004; Mead & Bower, 2000). Despite the lack of a consensus definition, person-centred care is agreed to be a multidimensional concept with a focus on the person's subjective experience of illness (Price, 2006). When integrated in daily care as a routine, person-centred care has been shown to have an advanced impact on patient and carer interaction, health outcomes, and satisfaction with care (Ekman et al., 2011).

Related to this principle, it has also been proposed that the environmental aspects of quality of life for patients affected by cancer include a strong focus on design, with examples such as windows with a view; acoustics that facilitate reflection, spiritual meditation, and intimate discussion; close links to nature; and sensory stimulation (Bakker et al., 2012; Tofle, 2009). A recent review of evidence-based design found that a conscious design adapted to patient needs had impact on a decrease in infection spreading, length of stay, pharmacological needs, and perceived stress among patients. Furthermore, symbolic objects found in the environment have been shown to have an impact on patients’ sense of self and well-being (Edvardsson et al., 2008; Peace, Holland, & Kellaher, 2005). These findings lead to the expectation that major considerations ought to be taken when designing healthcare environments to meet quality requirements, while considering patients’ needs and supporting patients’ sense of control, autonomy, and independence (Crews, 2005; Nahemow, 2000). In addition, it has been shown that when environmental stress exceeds the patient's ability to meet the demands, quality of life is adversely affected (Verbrugge & Jette, 1994). Furthermore, a recently published Cochrane review on environmental impact on health stressed the profound need for well-designed studies following intervention in healthcare environments (Drahota et al., 2012).

Based on these environmental prerequisites, it is evident that more knowledge is needed about what cancer patients value and consider important in the healthcare environment in order to support quality of life and quality of care, and to create a more person-centred environment in oncology care.

## Aim

The aim of this study was to describe what factors of the healthcare environment are perceived as being important to patients in oncology care.

## Methods

### Design

A qualitative design using focus group interviews was adopted. The study was part of a broader survey done earlier with an interest in various environmental aspects of oncology care.

### Patients and setting

Patients at two departments who had the experience of being treated at the oncology clinic on several occasions before and after the refurbishment of the physical environment were asked to participate in the focus group interviews. The patients in this study had therefore already been informed of the possibility of being asked to participate in future interviews in connection with completing the previous survey study about their experiences with the ward environment: the Person-Centred Climate Questionnaire (Edvardsson, Fetherstonhaugh, Nay, & Gibson, 2010). Eleven patients agreed to participate in further focus groups and were sent a letter with three alternative dates for focus group interviews along with patient information and consent forms. Based on their ability to participate, three focus group interviews were set up with patients (*n*=11). In the first and second focus groups, three patients participated in each group: two women and one man. In the third group, five patients participated: two men and three women. The mean age of the 11 participants was 54 years, in the range of 32–72 years. Two patients were diagnosed in 1970 and 1972, respectively, and the other nine patients were diagnosed between 2008 and 2010. The diagnoses were ovarian cancer (*n*=5), retinoblastoma (*n*=2), pulmonary cancer (*n*=1), breast cancer (*n*=1), and liposarcoma (*n*=1). All patients received chemotherapy and/or radiotherapy treatment prior to or during their participation in the focus groups.

The oncology clinic was under reconstruction at the time of the study, both the organization of the care and the physical settings in which the care was performed. The aim of these changes was to integrate inpatient and outpatient care in order to support flexibility and cooperation between professionals and between the different oncology specializations.

### Data collection

Focus group interviews are carefully planned discussions that take advantage of group dynamics for accessing rich information in an efficient manner (Kitzinger, 1994). Homogeneous focus groups are often recommended (Kitzinger, 1995), since participants who share certain experiences and perceptions often are more willing to exchange ideas and thoughts in a group. The rationale for using focus group interviews for data collection was the effectiveness of the method to explore people's knowledge and experiences. The ideal size for a focus group is between four and eight people (Kitzinger, 1994). Smaller groups have a benefit in that each person needs to play a prominent role, while in larger groups the opportunities to speak are more limited (Wibeck, Dahlgren, & Öberg, 2007). Although group interviews are often used simply as a quick and convenient way to collect data from several people simultaneously, focus groups explicitly use group interaction as part of the method (Kitzinger, 1995). This means that instead of the researcher asking each person to respond to a question in turn, people are encouraged to talk to one another: asking questions, exchanging anecdotes, and commenting on each other's experiences and points of view (Kitzinger, 1994).

The method is therefore particularly useful for exploring not only what people think but also how they think and why they think that way (Kitzinger, 1995; Stewart, Shamdasani, & Rook, 2007). In addition, group discussion can be particularly appropriate when seeking to encourage the participants to explore issues of importance to them, using their own vocabulary, generating their own questions, and pursuing their own priorities. In order to identify perceptions that contributed to achieving this aim, open-ended questions were asked that were directed at the participants’ experiences of the healthcare environment:

- What meaning do you attach to the concept *healthcare environment*?
- Can you give some examples of positive and/or negative aspects of the *healthcare environment* at the ward, and provide arguments to support your opinion?
- What do you consider to be important factors in *healthcare environments* in general, and why?

One researcher (M. B.) acted as a moderator and guided the discussion, and one researcher (I. K.) made detailed notes during each session. The three focus group interviews were conducted on three different occasions at the end of 2010 in a central and private location within the clinic. The interviews lasted between 60 and 90 min and were recorded on tape. They were transcribed verbatim immediately afterwards to minimize the difference between the verbal and written discourse.

### Ethical considerations

All participants received a letter describing the purpose and procedure of the study. The letter also stated that their participation was voluntary, that the study records would be kept confidential, and that their contributions would be unidentifiable in the final report. The Regional Ethics Committee in Gothenburg approved this study (Ref. no. 192-10).

### Content analysis

Focus groups are frequently subjected to content analysis, which is a research technique for making replicable and valid inferences from recorded speech and texts in the context of their use. The verbatim transcripts of the interviews were analysed using manifest content analysis, which is a well-known method for descriptive analysis of a text. The analysis was made to present the underlying meaning of the text, referred to as the latent conceptions from the interviews. Analysis was performed step by step in order to describe prominent categories (Krippendorf, 2004). The focus group interviews were read through several times by the first author (M. B.). Two other authors (I. K and H. W.) read a sample to obtain a sense of the totality. All interviews with narrations concerning different aspects of the physical and psychosocial environment at the oncology department were identified and extracted in one text. This text was divided into units of meaning, which were subsequently condensed and labelled with a code that adhered closely to the language used by the participants. These units of meaning were formed into subcategories and then into categories. All codes were checked back with the units of meaning, and the interview text and some codes were changed. This analysis was repeated several times. All codes were compared based on variations and similarities, and they were used to form tentative categories that were discussed and revised several times by the authors before they were finally arranged into three main categories and eight subcategories (Krippendorf, 2004). All four authors are registered nurses and researchers with varying experience of nursing care, and each participated in the process of reflection and discussion about the agreement of subcategories and categories.

## Results

Analysis of the patients’ perceptions of the environment at the oncology clinic, as expressed in the focus group interviews, forms a structure comprising three overall categories: *safety*, *partnership with the staff*, and *physical space*. The categories consist of eight subcategories, as presented in Table I. The three overall categories together with their subcategories are presented in this section, and they are illustrated with quotations from the interviews.

**Table I:** Adult patients’ perception of perceived important factors in the healthcare environment in oncology care.

Categories	Subcategories
Safety	Continuity and accessibility
	Privacy and community
Partnership with the staff	Being a person
	Participation and responsibility
	Communication
Physical space	Food and smell
	Visual impressions
	Surrounding sounds

### Safety

The three different categories include and focus on descriptions given by patients of their own perceptions of the environment. The category *Safety* consists of the subcategories *Continuity and accessibility* and *Privacy and community*.

#### Continuity and accessibility

For many patients, the importance of being cared for by the same staff throughout the disease process was associated with a sense of security. The patients argued that they needed to know who to contact at the ward when they were at home with a problem, but they also needed to feel confident and safe being cared for by someone who was fully aware of their situation. Those who did not attach great importance to the continuity of staff stated that it was not the person but the quality of the care that was crucial to their feeling safe as a patient. This finding is in contrast to the importance attached to seeing the same doctor during each care episode as well as experiencing good teamwork among the healthcare professionals. Teamwork was valued highly and was strongly linked to a sense of security. Accessibility of care, in terms of being able to reach the staff by phone at any time of the day or night, was also valued as part of a good caring environment:

Yes, it is easy to get hold of the nurse. You can get hold of someone almost 24 hours a day, and you can call the hospital ward at any time of day.

Patients expected that the healthcare professionals had forward planning in place, that they were briefed on what to do and why, and that they, in turn, would convey this information to the patients. The patients considered this process as integral to bolstering their sense of security and confirmation of their individual identities.

#### Privacy and community

One of the prerequisites identified by the patients for a safe, functioning healthcare environment is that it is spacious and inviting for both patients and staff. Other factors listed included the provision of beds and bedside tables that were modern and easy to use, and a kitchen and dining room that were functional and inviting. Having to wait for a bed on arrival at the clinic, or being greeted by patient beds stacked up in the corridor, was regarded as negative and frustrating. Many of the patients felt there was a lack of space for visitors during their treatment, and this was considered to be a very negative aspect.

When I'm here for my chemo, I want some privacy. It doesn't matter if I'm in the lounge watching TV or somewhere else … right now there are five people in here with IVs and …. You feel like you're on a bus during rush hour. I think it's difficult. If someone comes to visit, there's no room to sit. If all the patients had visitors at the same time, there wouldn't be enough room for chairs.

The new design of the clinic, providing large, spacious rooms for patients and where friends and family can sit and talk without disturbing other patients, was considered to be an extremely important improvement. Just as important as space for visitors was the availability of a space to withdraw to and be alone when necessary. The availability of single rooms with en suite toilets and showers was perceived by many as being very positive in terms of allowing them to choose to either be alone or socialize with others. Being forced to adhere to clinic routines—sleeping, eating, and living in close proximity to strangers when you are feeling vulnerable and exposed—was stated by several study participants to be both demanding and demeaning.

The only room that offers any privacy around here is the bathroom.

Being forced to encounter young people with cancer or to socialize with emaciated and frail patients in the dayroom was also considered to be difficult as it reminded the participants of their own declining health.

Untidy corridors and patient rooms were a source of great concern for the participants and made them feel uncertain. This element was especially crucial during the admission examination, which, for many, is the first encounter with oncology care and thus a strong predictor for their impression of the overall content and quality of care. A tidy, structured desk in the consultation room was associated with a feeling of security in relation to expectations of the care provided.

### Partnership with the staff

This category consists of the subcategories Being a person, Participation and responsibility, and “Communication.”

#### Being a person

To be treated well, feel cared for, feel welcome, and experience the time spent at the hospital as good and well structured were all considered to be important aspects of the healthcare environment. The first impression on arrival at a new healthcare unit, including the assurance of being in safe hands, was considered to be highly significant.

When I first came here I didn't care how it looked. … I was just bothered about the staff and how they treated me. Later, when I was receiving treatment and had to walk around the corridors, I started to notice how it looked. I would say that my first impression of this place was focused on how the staff treated me.

There was considerable emphasis on how the staff cared for the patients and the fact that small talk about everyday things was prioritized instead of focusing strictly on the disease, treatment, and medical records. The same applied to being treated as an equal. The impact of positive feedback and being noticed and listened to by the staff was considered to be crucial for the feeling of being seen as a unique individual and not just a patient. A good encounter was characterized by the staff taking time to talk, showing interest, and being positive and skilled.

… they remember who you are. Maybe they remember me because I have a rare form of cancer … but it doesn't feel that way. It feels like they remember me because of who I am.

This element also stressed the significance of being cared for by someone who demonstrates sincerity, empathy, and a “sure instinct.” Many patients appreciated an aesthetically pleasing environment, but this element could not compete with the overwhelming importance of the care culture. This culture was described as one that includes active and positive encounters with staff who were not afraid to be personally and professionally involved in their dealings with the patients.

Many of the patients had experienced numerous admissions at various hospitals and departments before the care episode at the oncology clinic, giving them the opportunity to compare settings. They described the atmosphere at the clinic to be special in terms of hospitality, good encounters, and person-centredness, and that it operated as an open climate and with plenty of time for questions. This element was what they missed most when being cared for at other care facilities.

I noticed the difference … a big difference. There wasn't the same warmth … sure it was professional, but something was missing.

#### Participation and responsibility

Transparency, good communication, and cooperation within the team were considered important factors when it came to feeling confident about care and treatment decisions. Respect by the staff for patient autonomy, supporting the patients in their actions, and being informed about alternative options were valued highly.

They listened to what you have to say and you're invited to be part of the decision process. I didn't like, for example, sharing a room, especially when you had to monitor your urine every day. So I stopped doing it and started to monitor my weight instead, which they thought was just as good. There was a dialogue between me and the staff about what was needed, and I really liked that.

Participation also involved patients’ personal responsibility for their health. It was considered important to be aware of and communicate subjective experiences and specific knowledge about being ill and, in doing so, influence the care that was being provided. There was also realization of the importance of keeping fit, exercising, sleeping, eating well, and trying to stay physically and mentally strong.

When you're at home, it is natural to move around and keep active but when you're admitted to hospital, there's no opportunity for physical activity.

Many of the patients, for physical and attitudinal reasons, found obstacles to maintaining a normal life pattern at the hospital very frustrating, since these conflicted with their desire to contribute to their good health.

I asked myself what I could do. I wanted to know how I could be stronger and what I could do for myself. No one ever helped me with that.

Participation and responsibility also included a desire to share experiences gained during the course of treatment in order to cope with fears and discomfort, and the design of the common areas was crucial to promoting or hindering these actions. A large day room supported these natural and spontaneous meetings with others, while a lack of common spaces, or spaces that were too small, led to many patients preferring to stay in their own room, thus missing out on the opportunity to interact with others.

Participation and responsibility also involved sharing experiences with loved ones. Having your relatives by your side as the disease progressed was, for many, both natural and necessary, while others chose to refrain from including family members. Whatever their preference was, the opportunity to choose and acceptance of their choice seemed to be the most important element for participants.

That is something they have probably not done, when I have been for different examinations and when I thought it was important to have a person with me, then I have taken him [husband] with me. There's not been anything strange, but he has not been invited specially by the staff.

Enough space for relatives to stay overnight, or for them to sit comfortably in the day room, was considered essential for them to want to stay and share everyday life with the patient. Being invited to help with the day-to-day care, as well as being informed about supportive interventions in which they could play an active part, were emphasized strongly.

#### Communication

Communication, often referred to as the “dialogue with the staff,” was highlighted as an important part of the caring culture, and was described as being either reciprocal or one-sided information—mostly the former. The patients often felt very well informed and never had to leave the hospital with unanswered questions. Both verbal and written information was considered satisfactory and could often be taken home, which promoted security and partnership. Conversely, some felt they had to find answers to lots of questions themselves. This triggered the ambition that the next time the doctor came, they would be well prepared with questions to verify the information. A recurring request was for “small talk,” expressed as encounters between two persons on equal terms, particularly in the dialogue with the doctor. The patients did not want to just listen to the doctor. They wanted to interact and discuss.

It is not just the information. It is important that they care and talk about other things and not just read from the patient's records.

In order to be heard, some of the patients stressed the importance of being known previously by the staff and developing a relationship. This was perceived as being very negative during first-time visits.

Nobody took me seriously when I wanted to talk about my problems because they didn't know me. It was like talking to a wall.

In contrast, being known by the staff mostly had the effect of promoting feelings of being listened to as an equal partner, and taking into account emotional and existential feelings as well as practical matters.

### Physical space

This category consists of the subcategories *Food and smell*, *Visual impressions*, and *Surrounding sounds*.

#### Food and smell

Considerable significance was attached to food and drink in connection with the overall experience of the healthcare environment, as well as to the physical space connected with those actions, for example the kitchen, which had to be clean, spacious, and fresh. The common experience of hospital food being dull, grey, and tasteless thus had a very negative influence. Overall, the lack of variety among the dishes and the absence of choice based on personal preference, coupled with the staff's shortcomings in being generous and flexible towards patients when it came to selecting or treating them with something homemade, had a strong impact on how the care culture was valued. Conversely, it was much appreciated if the staff took the time to provide information about what was good to eat and to make the meals attractive and the food more accessible, particularly during treatment for chemotherapy.

I seem to be one of the few to have had a good appetite all the time and I think that's pretty important. I would guess that food and the environment in the kitchen affect your appetite.

Smell was also claimed to be a significant part of the healthcare environment.

All that chemo makes you sensitive to smells and so on.

The smell when walking into the department and when the food trolleys arrive, as well as the smell of the staff (i.e., deodorant and perfume), all had meaning, which affected the patients. Good smell was associated with cleanliness. The availability of fresh air and the opportunity to open a window to inhale a good, outdoor smell or to air the room were all considered to be important elements of the healthcare environment.

#### Visual impressions

The first visual impression when visiting a ward was of great significance. A sense of harmony was perceived to be crucial for what to expect for the rest of the stay, including the ward atmosphere, design, temperature, and light.

Well, I saw it, the orange wall, as soon as I arrived. I thought it was very welcoming.

An environment that promotes a feeling of cleanliness, with bright, fresh rooms, was felt to be important and supported a feeling of well-being. At night, a common wish was to be able to make the room really dark, which could be difficult as large hospital windows easily let in light early in the morning. Another factor was the opportunity for tactile physical contact by the staff in the form of spontaneous hugs and pats on the shoulder or cheek, offering the patients confirmation that they were seen as a person, creating positive feelings and supporting good interaction.

The sincerity that you encounter here as a cancer patient. … Then came the biggest hug.

#### Surrounding sounds

Sounds within a healthcare environment were strongly associated with the ability to sleep and have a good night's rest, and control of excessive noise was regarded as essential to well-being. A quiet and calm environment at night was considered to be very important. Just as important as silence at night were everyday sounds during the day, such as natural sounds from the kitchen, music, or people chatting. Natural background sounds were really missed if they could not be heard.

It's very important when you're on 5-hour treatment that you have some amusement.

## Discussion

This study presents environmental factors that cancer patients value as crucial and important for the support of their well-being and functioning. The patients seem to perceive the care environment as a complex entity made up of several physical and psychosocial aspects. This complexity of experiences highlights the importance of not approaching the subject with the sole ambition of creating a “gold standard” for a good healthcare environment. Instead, improving the healthcare environment should incorporate a plan that allows for care to be tailored to the needs of the individual. Ultimately, the study of these environments should aim to learn more about what the patients consider to be important factors as a basis for a person-centred approach when creating person-centred healthcare environments.

The participants in this study were severely and chronically ill patients with long experiences of being in hospital. When they were asked about what, in their experience, were important aspects in healthcare environments, the physical aspects were not the most important; instead, psychosocial factors emerged strongly as the most important aspects of a care environment. The staff taking time to talk and showing interest in the patients, without being afraid of getting personally and professionally involved, were interpreted as being a sign of an empathic environment and supported a sense of empowerment and partnership. This is in line with earlier studies (Edvardsson et al., 2006), underlining the significance of seeing and being seen. The vital balance between privacy and community was stressed by the participants in our focus groups, where the opportunity to be on your own, to be a little private, was crucial. This element was also stressed in other studies, highlighting the risk of suffering if the private space is invaded. The importance of being involved in the planning of care is one of the main characteristics of a person-centred care approach (Ekman et al., 2011), which the participants in our study expressed in terms of being noticed and being listened to as a person and not just as a patient. Our findings are also in line with the study by McCormack, Karlsson, Dewing, and Lerdal. (2010), who emphasized the importance of getting to know the person behind the patient—who is the “expert in the illness experience”—in order to involve the person as an active partner in his or her care and treatment (McCormack et al., 2010).

The oncology ward in this study was described by the participants as a special place compared to their other experiences of healthcare environments, as described in earlier studies, for example by Edvardsson et al. (2006). Important factors mentioned in support of this notion were the well-developed teamwork, which provided a sense of security, as well as the forward planning conveyed to the patients. This observation lies firmly in line with the routines proposed to facilitate and safeguard person-centred care, with patient discussions to establish a partnership, as well as shared decision making and documentation of in-patient records to support the continuity and transparency of the provider–patient partnership (Ekman et al., 2011). The possibility of participating in and being responsible for your own care was considered to be important by the participants, along with the opportunity to choose whether they preferred socializing with others or withdrawing in solitude. The beneficial effect of being able to choose has also been highlighted by elderly persons in nursing homes (Falk, Wijk, & Persson, 2011), who state that being allowed the use of private space is an important means of escaping from stressful moments, even if it was only for a while (Edvardsson et al., 2006).

Many of the participants in our study valued the opportunity for privacy that only a single room could offer. There are several studies showing the beneficial effect of being alone. Lawson, Phiri, and Wells-Thorpe (2003) found that patients who had single rooms were more satisfied with their stay and with the hospital environment than those who remained in an open ward. A study by Dijkstra et al. (2006) also emphasized that seclusion had a positive effect on patient health and well-being. Even communication between the patient and the staff has been seen to improve if the patient has a single room (Ulrich, Zimring, Joseph, Quan, & Choudhary, 2004).

Other physical aspects of the healthcare environment that were highlighted in this study as being important to the patients were food and smell, and visual and sound impressions, which have considerable impact on the health and well-being of the patient. The change in taste and smell sensitivity among patients receiving chemotherapy is a well-known effect (Bernhardson, Tishelman, & Rutqvist, 2009; Duffy, Fast, Lucchina, & Bartoshuk, 2002), and these have an impact on the patient's day-to-day life (Bernhardson et al., 2009). This knowledge, together with the description from this study of the importance of fresh air and being able to inhale good, outdoor smells, demonstrates the importance of an environment that offers the chance to open a window. The smell when walking into the department, the smell when the food trolleys arrive, and, most importantly, the smell of the staff (i.e., deodorant and perfume) are important factors to consider in a patient-centred environment. Less important to the participants was whether the surroundings were luxurious and whether they contributed to a good atmosphere. In line with studies by Edvardsson et al. (2006), first impressions were not essential to how participants felt about their quality of care as their treatment progressed.

### Strength and limitations

We are aware of the fact that we cannot say to what degree this small sample is representative of other patients in oncological departments, but the aim of the study was to get in-depth knowledge from patients about what factors of the healthcare environment patients in oncology care perceived as being important. A strength of our sample is that it is likely that we had a relatively well-experienced sample of people who volunteered to participate in this study about the environmental experiences. Typically, focus groups consist of between six and 12 members drawn from a study population of interest (Stewart et al., 2007). As we thought that focus groups and group processes can help people to explore and clarify their views and attitudes efficiently and place particular importance on interaction between participants, we decided to implement these in spite of the small sample. Also, despite the small number of participants in this study, it is known that when the topic is emotionally charged or sensitive, smaller groups of about five participants are recommended (Cote-Arsenault & Morrison-Beedy, 1999). Nevertheless, future studies with more participants are needed in order to explore these results further.

## Conclusion

Both physical and psychosocial aspects of the environment seem to have a strong impact on patients’ well-being and functioning. Even though psychosocial aspects were of the greatest importance in this study, helping the patient to feel confident and secure with the care provided and physical aspects of the environment were also stressed as having a significant impact on managing and respecting the special needs and symptoms associated with cancer care.

The physical and organizational reconstruction of the oncology clinic where the focus groups interviews were conducted aimed to support flexibility and cooperation between staff, and it adapted the physical organization to support a person-centred approach. According to the findings of this study, the aim of the reconstruction is in close agreement with what the patients claimed to be essential for a person's well-being and functioning. The take-home message from this outcome is to put maximum effort into viewing each patient as a unique person, establishing opportunities for good encounters between staff and patients, and supporting these activities with a physical environment that has sufficient space, not only to socialize with staff, fellow patients, and family members but also to withdraw and rest.
